# Gunshots through laminated glass: expelled compounded fragments as a function of bullet type

**DOI:** 10.1007/s00414-022-02904-z

**Published:** 2022-11-16

**Authors:** Constantin Lux, Alexander Krutzek, Tobias Reich, Stephan Welkerling, Jan M. Federspiel, Frank Ramsthaler, Hannes Gruber, Patrick Sauer, Natascha Kern, Marcel A. Verhoff, Mattias Kettner

**Affiliations:** 1 Institute of Legal Medicine, University Hospital of Frankfurt, Goethe University, Kennedyallee 104, 60596 Frankfurt/Main, Germany; 2 Meadow Bridge Training Center, Außerhalb 3, 65468 Trebur, Germany; 3 Hessisches Landeskriminalamt, Hölderlinstr. 1-5, 65187 Wiesbaden, Germany; 4 Institute of Legal Medicine, University Hospital of Homburg/Saar, Homburg, Germany

**Keywords:** Forensic ballistics, Reconstruction, Laminated glass, Intermediate target, Expelled fragments, Wounding potential

## Abstract

In the frame of an experimental setting, the formation of round-shaped compounded glass fragments on the exit site after gunshots through a windshield was examined. For that purpose, a 9 × 19 mm pistol (HK P30) and two different cartridges containing (a) a full metal jacketed round-nosed projectile and (b) a deformation projectile were used. On the basis of 52 gunshots, the morphology, impact angles and terminal ballistics of occurring compounded glass fragments were examined. The results showed that the compounded glass fragments’ morphology allowed for the differentiation of two used projectiles. Fragments were able to cause round-shaped defects in a single cotton layer (T-shirt) with subsequent penetration of up to 2.4 cm into ballistic gelatin (10%, 4 °C). As a function of the projectile type, the compounded glass fragments showed different reproducible impact angles that differed notably from the known conical pattern of expelled glass fragments after bullet penetration. These findings might help to explain the atypical morphology of gunshot wounds with laminated glass as an intermediate target and prevent possible misinterpretations when reconstructing the sequence of events.

## Introduction

In gunshot-associated crimes, the reconstruction of the sequence of events can be challenging if no reliable testimonies or documentary footage is available, especially when the crime scene has been manipulated or a gunshot wound does not lead to immediate incapacitation of the victim [1–3]. In these cases, finding and understanding gunshot-related traces may be crucial for legal assessment. As an intermediate target in the line of fire, glass offers some features that may allow for reconstructing the spatial relation between the shooter and the victim at the moment a shot was fired [3, 4]. Thus, the behaviour of bullets and expelled glass fragments during projectile penetration has been examined in the past [4–8]. Glass fragments expelled from the exit site of the penetrated glass in the course of bullet transition typically spread out in a conical pattern at right angles to the plane of the glass. When these glass particles strike human skin, the resulting injury pattern consisting of numerous finely dispersed superficial dermal lesions bears resemblance to the typical stippling caused by unburnt powder grains when a shot is fired from near distance [9] and is referred to as “pseudo stippling”. The topographical distribution of the injury pattern may reveal aspects of the body posture of the victim in relation to the location of the bullet penetration through glass when the shot was fired [3, 4]. With increasing distance between the skin and glass as intermediate target, the wounding potential of expelled glass fragments decreases rapidly [4].

For the majority of modern windshields and side-windows in some cars, as well as windows in buildings, laminated glass is used. It usually consists of at least two layers of glass with an interlayer of plastic film, mostly composed of polyvinyl butyral (PVB), ethylene–vinyl acetate (EVA) or a thermoplastic elastomer (TPE). With regard to safety issues, the interlayer is supposed to keep the glass layers bonded during and after breakage and prevent the glass from breaking up into large, sharp and potentially harmful pieces. Further benefits of these interlayers are defined by particular properties of the material used and include UV screening, damping of sound and temperature isolation [10].

In a case of homicide, laminated glass as an intermediate target led to an irregular, atypical wounding pattern [3]: a woman was found lying on the street in front of her car. External examination revealed shot wounds of the neck region. Upon inspection of the car, the driver’s door was open and a bullet hole was identified in the driver’s side window consisting of laminated glass. Next to the car, a cartridge case calibre 0.32 ACP was found.

**Fig. 1:**
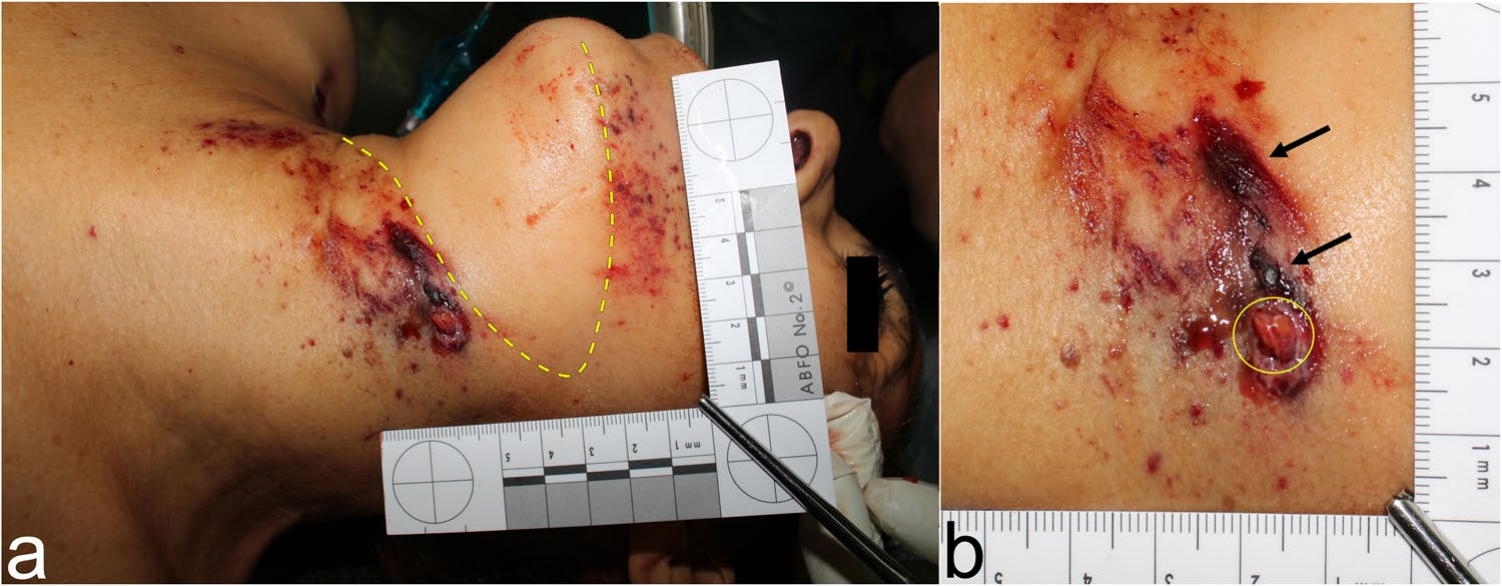
**a** Entry site of the bullet with dense pseudo stippling and demarcated interruption (*yellow striped line*). **b** Magnification of the entry wound (*yellow circle*) and deep dermal abrasions (*black arrows*)

Autopsy revealed an entry wound on the left side of the neck with dense pseudo stippling due to expelled glass fragments. Amid the pseudo stippling pattern, a sharply demarcated interruption of the otherwise “spray shadow”-like pattern was found (Fig. 1a). Upon reconstruction, this void had been caused by the left mandible due to a slight rotation of the victims’ head to the left and downwards when sitting in the drivers’ seat at the moment of firing. Close to the entry wound, the skin showed a circumscribed, deep dermal abrasion (Fig. 1b). As the intact projectile was secured between layers of clothing, fragmentation of the projectile could be excluded as a reason for the deep abrasion pattern.

To understand the general mechanism leading to that wound morphology, in particular the deep dermal abrasion pattern, experimental gunshots were fired through laminated glass (windshield) using a 9 × 19 mm cartridge loaded with a round-nosed full metal jacketed (FMJ) bullet. In addition to the expected fine glass particles spreading out in a conical pattern at right angles to the plane of the glass, an expelled round-shaped compounded glass fragment of about 1 cm in diameter was observed. To further characterise dispersed and compounded glass fragmentisation, three different experimental settings were examined in the present study:

1. Identification and morphological characterisation of round-shaped compounded glass fragments expelled after gunshots through laminated glass (*n* = 10).
2. Trajectory angles of round-shaped compounded glass fragments calculated from resulting defects at distances of 35 cm and 70 cm behind the intermediate target (windshield) (*n* = 20).
3. Examination of traces upon contact with a single cotton layer (T-shirt) as well as subsequent penetration depths into ballistic gelatin at distances of 35 cm and 70 cm behind the intermediate target (windshield) (*n* = 12). Furthermore, a skin surrogate was used to examine the compounded glass fragments’ potential to perforate skin at a distance of 70 cm behind the windshield (*n* = 10).

Thereby, the following objectives were analysed:

• Primary: Can we discriminate the usage of different projectile types based on distinct parameters of the employed projectile and the resulting expelled compounded glass fragment, namely the horizontal and vertical deviation of the glass fragment from the perpendicular of the windshield surface?
• Secondary: Can we discriminate the usage of different projectile types based on the dimensions and/or morphology of the expelled compounded glass fragment?
• Tertiary: Are round-shaped compounded glass fragments able to penetrate textile or skin simulant?

## Materials and methods

Two different 9 × 19 mm projectile types were used: One is a deformation projectile commonly used by German police officers, and the other is a commercially available round-nosed full metal jacketed (FMJ) projectile. Each shot was fired through a windshield mounted with an angle of 15°, measured in the centre of the windshield, resembling a typical car windshield angle.

### Weapon, ammunition and windshield

For all shooting experiments, a 9 × 19 mm P30 pistol (Heckler & Koch, Oberndorf am Neckar, Germany) was used to fire shots with two different 9 × 19 mm cartridges containing a deformation (1) and a round-nosed full metal jacketed (2) bullet:

1. PEP 2.0 (MEN—Metallwerk Elisenhütte GmbH, Nassau, Germany)—projectile weight: 95 g
2. 9 × 19 mm LUGER FMJ (Prvi Partizan, Užice, Serbia)—projectile weight: 124 g

As the intermediate target, a windshield (Hyundai, Seoul, South Korea) made of laminated glass (Saint-Gobain Sekurit, Paris, France) with a thickness of 4.9 mm and a PVB interlayer was used.

### 3D documentation of the windshield surface

The curvature of the windshield was measured using the FARO Focus S150 3D-laser scanning system (Faro Europe GmbH, 70,825, Korntal-Münchingen, Germany), which utilises terrestrial laser scanning technique as an active, non-contact optical polar measurement method to digitally reproduce complex three-dimensional (3D) object surfaces. The result of the scanning process is an arranged regularly 3D point cloud, which can then be used for the extraction of measurements, to generate 2D line maps or complex 3D models, visualisations and deformation analyses. The principle of this method is based on reflector-less distance measurement with recording of horizontal and vertical angle. The emitted laser beam is deflected by means of a rotating polygon mirror to the surface of an object, reflected and received again on the scanner. Results are 3D coordinates of the recorded surface calculated from the distance and angles for each point. However, the reconstruction of surface geometry for transparent objects is complicated by the fact that light is transmitted through, refracted and in some cases directionally reflected by the surface. To resolve both, the problem of transparency of the surface and the specular reflection, coating material was applied to the surface of the windshield, which was then spatially digitised with a 3D resolution (3D point cloud) of approx. 1 mm.

### Setting 1: Morphology and fracture pattern of round-shaped compounded glass fragments (Table 1)

**Table 1:** Results of setting 1 with weight, maximum and minimum diameters (*d*) as well as fracture morphology of resulting compounded glass fragments. PEP: 9 × 19 mm “Polizei Einsatz Patrone”, loaded with a deformation projectile; FMJ: 9 × 19 mm cartridge loaded with a full metal jacketed round-nosed projectile

		Compound glass fragment			
Gunshot no	Cartridge/projectile	Weight [g]	*d* min [mm]	*d* max [mm]	Fracture pattern
1	PEP	0.127	12.33	12.9	Diffuse
2	PEP	0.144	10.51	12.4	Diffuse
3	PEP	0.133	11.03	11.37	Diffuse
4	PEP	0.112	10.87	11.2	Diffuse
5	PEP	0.154	10.22	12.55	Diffuse
6	FMJ	0.142	11	11.74	Radial
7	FMJ	0.121	9.76	10.43	Radial
8	FMJ	0.103	8.67	10.82	Radial
9	FMJ	0.091	9.76	10.65	Radial
10	FMJ	0.064	9.04	10.41	Radial

To study the morphology and to analyse the pattern of expelled round-shaped compounded glass fragments, 10 gunshots (*n* = 5 per ammunition type) were fired horizontally through a windshield mounted with an angle of 15° (measured in the centre) to the vertical axis. The distance between the muzzle and windshield (D1) was 175 cm. Compounded glass fragments within a 2.5-m radius were collected and weighted. Minimum and maximum diameters were measured using a digital calliper (measurement in mm rounded to the first decimal; WorkZone GmbH, Hamburg, Germany). The fracture patterns of the round-shaped compounded glass fragment’s surface were photographically documented.

### Setting 2: Impact angles of the round-shaped compounded glass fragments (Fig. 2)

**Fig. 2:**
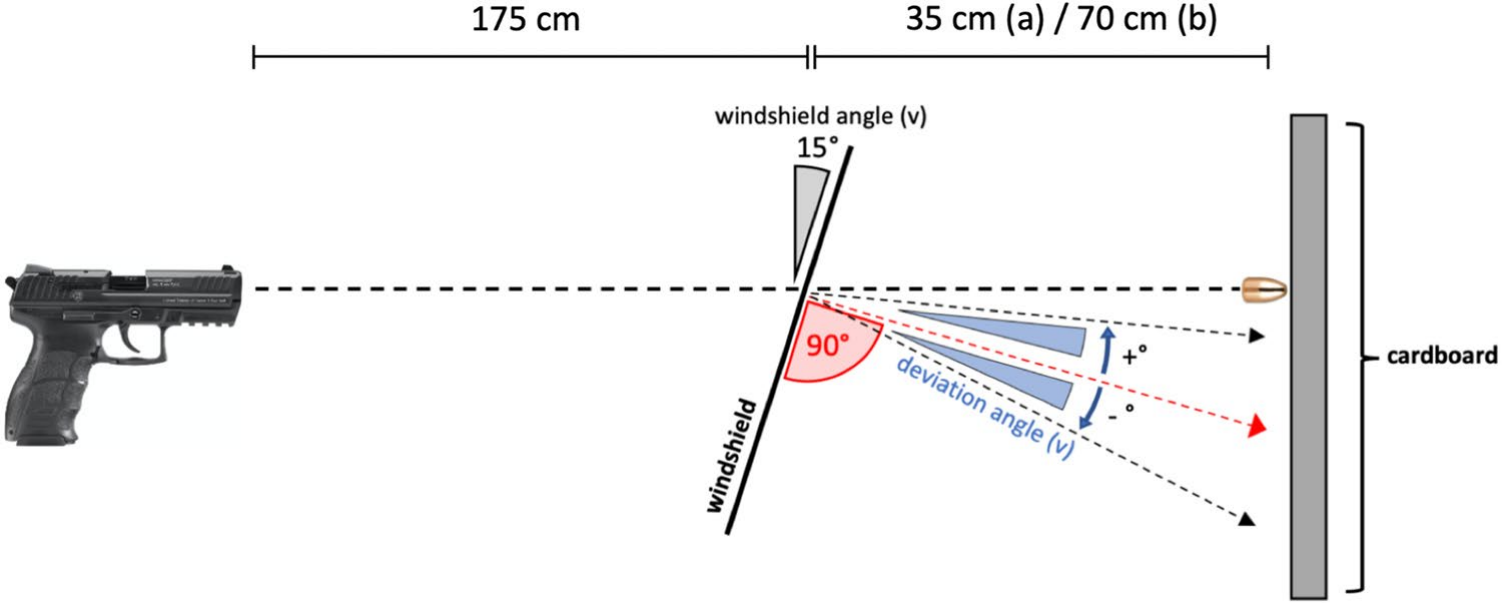
Schematic illustration of experimental setting 2

In total, 20 gunshots (*n* = 10 per ammunition type and *n* = 5 per distance between intermediate and terminal target) were fired horizontally through a windshield. The distance between the muzzle and windshield (D1) was 175 cm. In the horizontal bore axis, 35 cm (group a; *n* = 5 per ammunition type) and 70 cm (group b; *n* = 5 per ammunition type) behind the windshield (D2), a 45 × 35 cm and 0.5-mm-thick paper cardboard was positioned aligned to the vertical axis. For each shot, the aiming point and centres of impact locations of the projectile as well as of the compounded glass fragment were measured. The impact angles of compounded glass fragments as well as their horizontal and vertical deviation from a line lying in the right angle to the plane of the glass were calculated. These parameters were defined as vertical (v) and horizontal (h) deviation angle. Vertical deviation angles above the right angle to the plane of the windshield surface were defined as positive, beneath as negative vertical deviation angles. Horizontal deviation angles to the left side from the shooter’s point of view were defined as negative, to the right side as positive horizontal deviation angles. The shots were fired at room temperature (18 °C) and a humidity of 60% in a closed shooting range without air draft or weather influences. The velocity of projectiles was measured using the photoelectric sensor technology method (BMC 18, W. Mehl, Diebach, Germany).

### Setting 3 (Fig. 3)

**Fig. 3:**
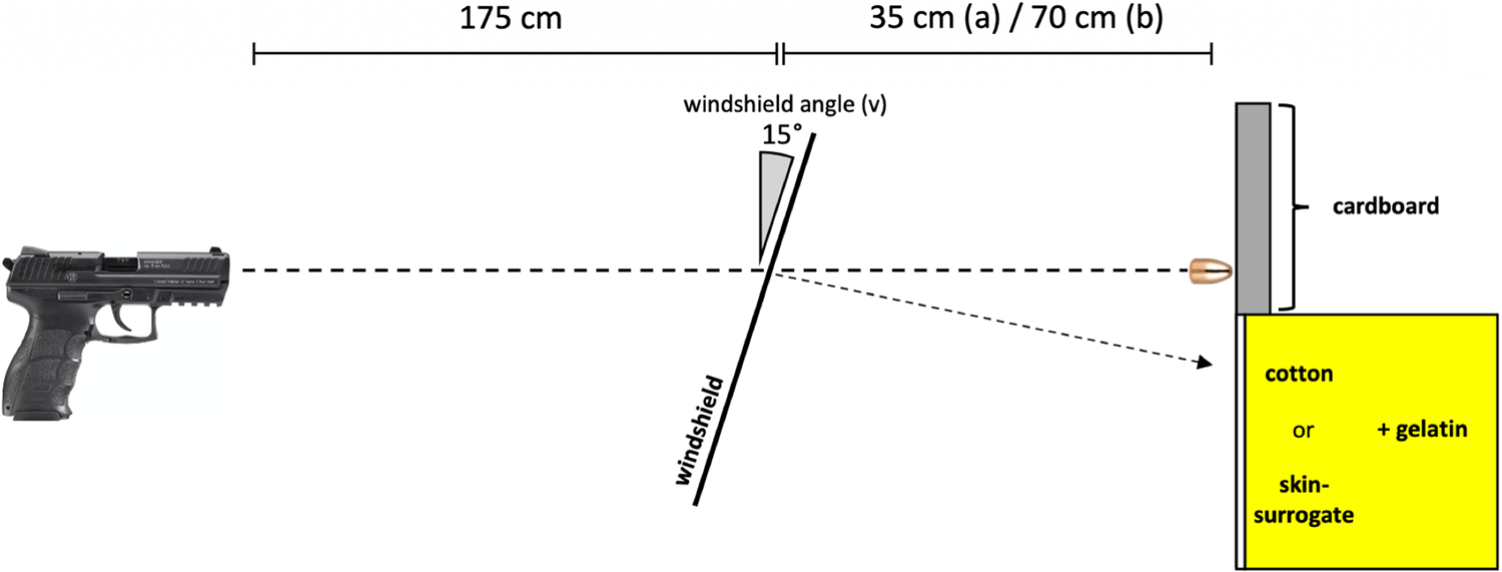
Schematic illustration of experimental setting 3

- Perforation of cotton and penetration depth in gelatin
  Twelve gunshots (*n* = 6 per ammunition type) were fired through a windshield under the same conditions as in setting 2. Composite models made of a single layer of 10 × 10 cm white cotton (Lacoste, Paris, France) and 10 × 10 × 10 cm blocks of 10% ballistic gelatin (Ballistic 3, Gelita, Eberbach, Germany) manufactured following Facklers’ instructions [11] and cooled down to 4 °C were used as targets for expelled compounded glass fragments and positioned at the same distances as the card boards in setting 2 (35 cm, *n* = 3 per ammunition type; 70 cm, *n* = 3 per ammunition type). Diameters of defects in the cotton layer as well as penetration depths of compounded glass fragments in gelatin were measured. The fragments of laminated glass were extracted manually, weighed and photographed. Smaller glass particles were filtered out of melted down gelatin using coffee filter bags (Melitta Europa GmbH & Co. KG, Minden, Germany).
- Perforation of skin surrogate

A skin surrogate was used similar as described by Bir et al. [12] using a 6-mm-thick layer of ethylene-vinylacetat-copolymer foam sheet covered by a 1.4-mm-thick layer of natural chamois strapped on 10 × 10 × 10 cm blocks of 10% ballistic gelatin (4 °C). For preliminary evaluation, a pump air rifle (881 Daisy Powerline Air Rifle, Daisy Outdoor Products, Rogers, USA) and 0.53 g 0.177 lead round ball ammunition was used (*n* = 70).

With each cartridge, 5 shots each were fired through the windshield. Skin surrogates were adjusted 70 cm behind the windshield, and expelled compounded glass fragments were caught. Penetration of the skin surrogate and, if applicable, penetration depth into gelatin were measured.

### Statistical analysis

Statistical analysis was performed using the statistical software package MedCalc (Version 20.104 MedCalc Software Ltd., Ostend, Belgium). Continuous variables were described using mean and appropriate measures of variation (e.g. standard deviation (SD), minimum or maximum), whilst nominal variables were described using absolute frequencies. For the inductive statistics, a significance level of alpha = 0.05 was defined. Thus, *p* values below 0.05 were assumed to be statistically significant. Comparisons used the non-parametric Mann–Whitney *U* test. Because of limited sample size (*n* ≤ 20), the exact probability was calculated [13]. Additionally, based on type-I- and type-II-error assessment per comparison, the required number of experiments for reliable statistical test results was calculated [14]. In these calculations, an alpha = 0.05 and a beta = 0.1 were used. Adjustment of the *p* values was done using the Bonferroni method.

## Results

### 3D scanning of the windshield surface

Local planes were calculated in the close-up area around each individual shot damage using selected 3D coordinates of the measured windshield surface. The vector normals of the individual local planes corresponded approximately to the orthogonal directions, relative to the windshield surface. Thus, the origin of the respective orthogonal direction was located at the centre of the corresponding shot defect. The calculated normal vectors of the individual shot defects were converted into corresponding horizontal and vertical angles related to the underlying coordinate system. The results for setting 2 are shown in Tables 2.

**Table 2:** Results of setting 2. PEP: 9 × 19 mm “Polizei Einsatz Patrone”, loaded with a deformation projectile; FMJ: 9 × 19 mm cartridge loaded with a full metal jacketed round-nosed projectile; V1: projectile velocity (measured at a distance to the muzzle of 1 m); E1: projectile energy (measured at a distance to the muzzle of 1 m); D2: distance between windshield and cardboard; windshield angle: angle of the windshield at the bullet impact site; v: vertical deviation angle; h: horizontal deviation angle

					Windshield angle [°]		Deviation angle [°]	
Gunshot no	Cartridge	V1 [m/s]	E1 [J]	D2 [cm]	v	h	v	h
1	PEP	410	517	35	16.2	− 1.9	6.4	5.5
2	PEP	414.7	529	35	16.1	− 0.5	6.4	2.4
3	PEP	407.3	511	35	16.1	2.9	9.1	− 1.7
4	PEP	411.5	521	35	16	1.6	8.1	1.9
5	PEP	412.8	525	35	16.5	3.9	9.7	− 0.8
6	PEP	408.9	515	70	17.8	6.3	6.3	− 1.9
7	PEP	408.6	514	70	17.8	4.9	8.1	− 1.4
8	PEP	410.4	518	70	16.7	5	5.5	− 1.3
9	PEP	x	x	70	18.8	4.9	4.1	− 0.5
10	PEP	407.9	512	70	18.9	7.2	4.3	0.3
11	FMJ	x	x	35	15.9	0.4	11.8	0.8
12	FMJ	331.9	443	35	15.6	1.6	12.5	− 2.5
13	FMJ	328.8	434	35	16.4	5.5	14.1	− 4.2
14	FMJ	x	x	35	15.5	5.5	11.4	− 6.1
15	FMJ	x	x	35	15.7	6	11.9	− 6.3
16	FMJ	320.2	412	70	17	1.6	15	− 3.3
17	FMJ	326.7	429	70	17	0	15.9	− 1.6
18	FMJ	330	438	70	17.4	− 1	16.7	− 0.3
19	FMJ	x	x	70	16.7	− 0.5	14.9	− 2.4
20	FMJ	x	x	70	16.7	− 1.9	15.9	1.9

### Setting 1 (Table 1; Fig. 4)

**Fig. 4:**
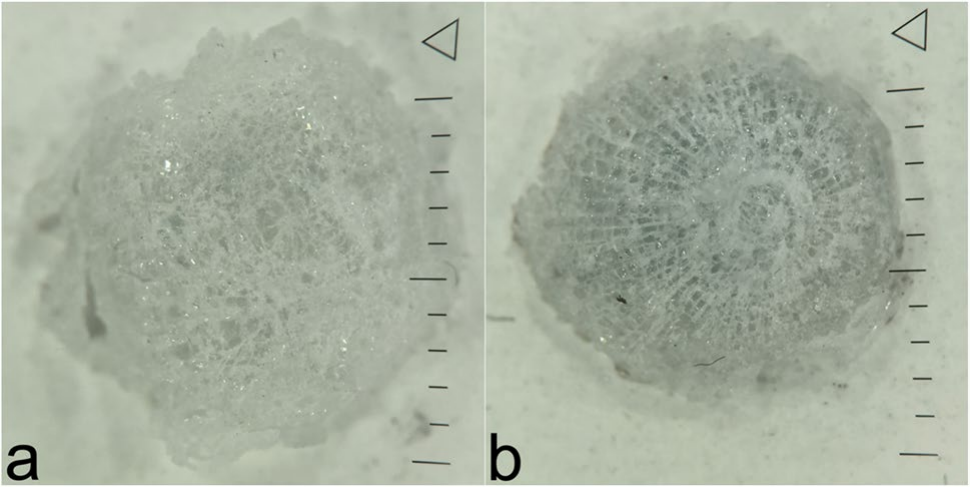
a Compound glass fragment after penetration of the windshield by a deformation projectile (PEP 2.0; MEN) with rather diffuse fracture pattern compared to b. b Compound glass fragment after penetration by a full metal jacketed projectile under comparable conditions with a central, round-shaped, prominent fracture zone of 2–3-mm diameter followed by a radiate fracture pattern reaching the margin of each fragment

#### Morphology, measures and weight of compounded glass fragments

Of all 10 gunshots, each resulted in a round-shaped, slightly curved compounded fragment of plastic film (former PVB interlayer) with firmly indented, fine fragments of glass on the convex side. Compounded glass fragments after shots with the deformation projectile (cartridge 1; *n* = 5) resulted in a rather diffuse, but similar and thus comparable fracture morphology. These were clearly distinguishable from fragments expelled after shots fired with the round-nosed FMJ projectile (cartridge 2; *n* = 5) showing a central, round-shaped, prominent fracture zone of 2–3 mm diameter followed by a radiate fracture pattern reaching the margin of each fragment.

The mean weight of compounded glass fragments retrieved from gunshots fired with deformation bullets was 0.134 g (SD 0.016). Minimum diameters showed a mean value of 10.9 mm (SD 0.81), and maximum diameters a mean value of 12.1 mm (SD 0.75).

Fragments collected after gunshots fired with the FMJ projectile weighed less with a mean weight of 0.104 g (SD 0.029) and showed smaller diameters (minimum: 9.6 mm, SD 0.89; maximum 10.8, SD 0.89).

### Setting 2 (Table 2)

#### Deviation angles of trajectories of compounded glass fragments—PEP 2.0 (1)

*Vertical deviation angles* of compounded glass fragments expelled when deformation projectiles were shot through the windshield (*n* = 10; a + b) ranged from a minimum of 4.6° to a maximum of 9.7°, with a mean value of 6.8° (SD 1.91°). For group a (*n* = 5; D2 = 35 cm), a minimum of 6.4° and a maximum of 9.7° with a mean value of 7.9° (SD 1.52°) were measured. For group b (*n* = 5; D2 = 70 cm), a minimum of 4.1° and a maximum of 8.1° with a mean value of 5.7 (SD 1.63) were measured.

The *horizontal deviation angles* in total (*n* = 10; a + b) showed a minimum of − 1.9° and a maximum of 5.5° with a mean value of 0.6 (SD 2.36). For group a (*n* = 5, D2 = 35 cm), a minimum of − 1.7° and a maximum of 5.5° with a mean value of 1.5° (SD 2.85°) were calculated. Group b (*n* = 5, D2 = 70 cm) showed a minimum of − 1.9° and a maximum of 0.3° with a mean value of − 0.9° (SD 0.87°).

#### Deviation angles of trajectories of compounded glass fragments—round-nosed FMJ (2)

*Vertical deviation angles* of compounded glass fragments expelled from the windshield during penetration by round-nosed FMJ projectiles (*n* = 10; a + b) ranged from a minimum of 11.4° to a maximum of 16.7°, with a mean value of 14° (SD 1.96°). For group a (*n* = 5; D2 = 35 cm), a minimum of 11.4° and a maximum of 14.1° with a mean value of 12.3° (SD 1.06°) were measured. For group b (*n* = 5; D2 = 70 cm), a minimum of 14.9° and a maximum of 16.7° with a mean value of 15.68 (SD 0.74) were measured.

The *horizontal deviation angles* (*n* = 10; a + b) showed a minimum of − 6.3° and a maximum of 1.8° with a mean value of − 2.4° (SD 2.72). For group a (*n* = 5, D2 = 35 cm), a minimum of − 6.3° and a maximum of 0.8° with a mean value of − 3.6° (SD 2.94°) were measured. Group b (*n* = 5, D2 = 70 cm) showed a minimum of − 1.9 and a maximum of 0.3° with a mean value of − 0.9° (SD 0.87°).

### Setting 3 (Fig. 5)

**Fig. 5:**
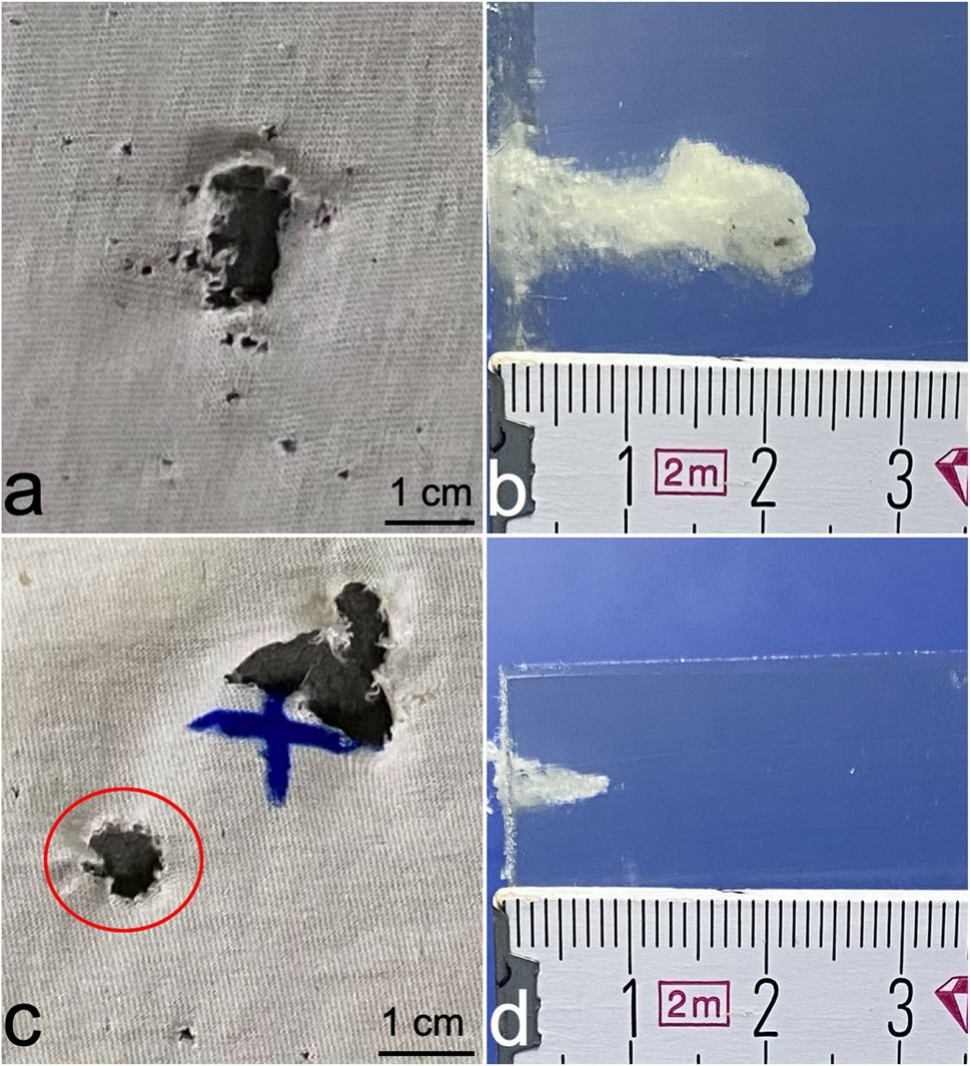
Exemplary results of setting 3 at distances of 70 cm between windshield and cotton. a, b Cotton defect (a) and gelatine (b) after penetration by a compound glass fragment (PEP; gunshot no. 4). c, d Corresponding findings after firing of a full metal jacketed projectile (gunshot no. 10). c: The round-shaped cotton defect marked by a red circle is caused by the compound glass fragment, the bigger, rather irregular defect by the bullet. The blue cross indicates the aiming point

- Perforation of cotton (Table 3)
  In all 12 shots, compounded glass fragments perforated the cotton layer leading to round-shaped defects and partially irregular, ragged margins. Diameters of defects caused by fragments deriving from shots using a deformation projectile (cartridge 1; *n* = 6) were generally bigger with a minimum of 14.6 mm, a maximum of 25.3 mm and a mean value of 19.5 mm (SD 3.87) than those resulting from shots fired with FMJ (cartridge 2; *n* = 6) showing a minimum of 8.4 mm, a maximum of 16.4 mm and a mean value of 12.2 mm (SD 2.88).
  With a distance (D2) of 35 cm between windshield and composite models, mean diameters of the cotton defects were bigger for both projectiles with 22.2 mm (cartridge 1; *n* = 3; SD 2.76) and 14.3 mm (cartridge 2; *n* = 3; SD 2.1) as compared to 16 mm (cartridge 1; *n* = 3; SD 7.21) and 10 mm (cartridge 2; *n* = 3; SD 1.65) at a distance of 70 cm (D2).
  When the deformation projectile (cartridge 1; *n* = 6) was used, penetration depths from 10 mm (min) to 24 mm (max) with a mean value of 16.5 mm (SD 4.63) were observed. Shots using the round-nosed FMJ projectile (cartridge 2; *n* = 6) resulted in penetration depths ranging from 4 mm (min) to 14 mm (max) with a mean value of 9.8 mm (SD 3.82).
  At a distance (D2) of 35 cm between windshield and composite models, mean values of perforation into gelatin were bigger for both projectiles with 22.2 mm (cartridge 1; *n* = 3; SD 2.76) and 14.3 mm (cartridge 2; *n* = 3; SD 2.1) as compared to mean values of 16 mm (cartridge 1; *n* = 3; SD 7.21) and 10 mm (cartridge 2; *n* = 3; SD 1.65) with a distance of 70 cm.
- Perforation of skin surrogate (Table 4)

**Table 3:** Results of setting 3 for textile penetration (cotton) and subsequent penetration of ballistic gelatine resembling soft tissue. PEP: 9 × 19 mm “Polizei Einsatz Patrone”, loaded with a deformation projectile; FMJ: 9 × 19 mm cartridge loaded with a full metal jacketed round-nosed projectile; D2: distance between the windshield and cardboard

Gunshot no	Cartridge	D2 [cm]	Material	Cotton defect diameter [mm]	Penetration depth in gelatine [mm]
1	PEP	35	Cotton + gelatin	21.1	17
2	PEP	35	Cotton + gelatin	20.1	16
3	PEP	35	Cotton + gelatin	25.3	18
4	PEP	70	Cotton + gelatin	14.6	24
5	PEP	70	Cotton + gelatin	15.8	14
6	PEP	70	Cotton + gelatin	20.3	10
7	FMJ	35	Cotton + gelatin	12.2	13
8	FMJ	35	Cotton + gelatin	16.4	14
9	FMJ	35	Cotton + gelatin	14.3	8
10	FMJ	70	Cotton + gelatin	10	8
11	FMJ	70	Cotton + gelatin	8.4	12
12	FMJ	70	Cotton + gelatin	11.7	4

**Table 4:** Results of setting 3 using skin surrogate and ballistic gelatine. PEP: 9 × 19 mm “Polizei Einsatz Patrone”, loaded with a deformation projectile; FMJ: 9 × 19 mm cartridge loaded with a full metal jacketed round-nosed projectile

	Penetration	
Projectile	Skin surrogate	Gelatine [cm]
PEP	Yes	0.3
PEP	Yes	0.2
PEP	Yes	0.5
PEP	No	0
PEP	Yes	0.7
FMJ	No	0
FMJ	No	0
FMJ	No	0
FMJ	No	0
FMJ	No	0

In the frame of preliminary shooting tests, round lead balls with velocities above 116.5 m/s and a corresponding energy density of 22.64 J/cm^2^ were able to penetrate the skin surrogate and into ballistic gelatin.

Four of 5 compounded glass fragments generated after shots with the 9 × 19 mm deformation projectile (cartridge 1) were able to penetrate the skin surrogate and up to 0.7 cm into ballistic gelatin. Using the 9 × 19 mm FMJ round-nosed projectiles (cartridge 2), expelled compounded glass fragments were not able to perforate the skin surrogate.

**Fig. 6:**
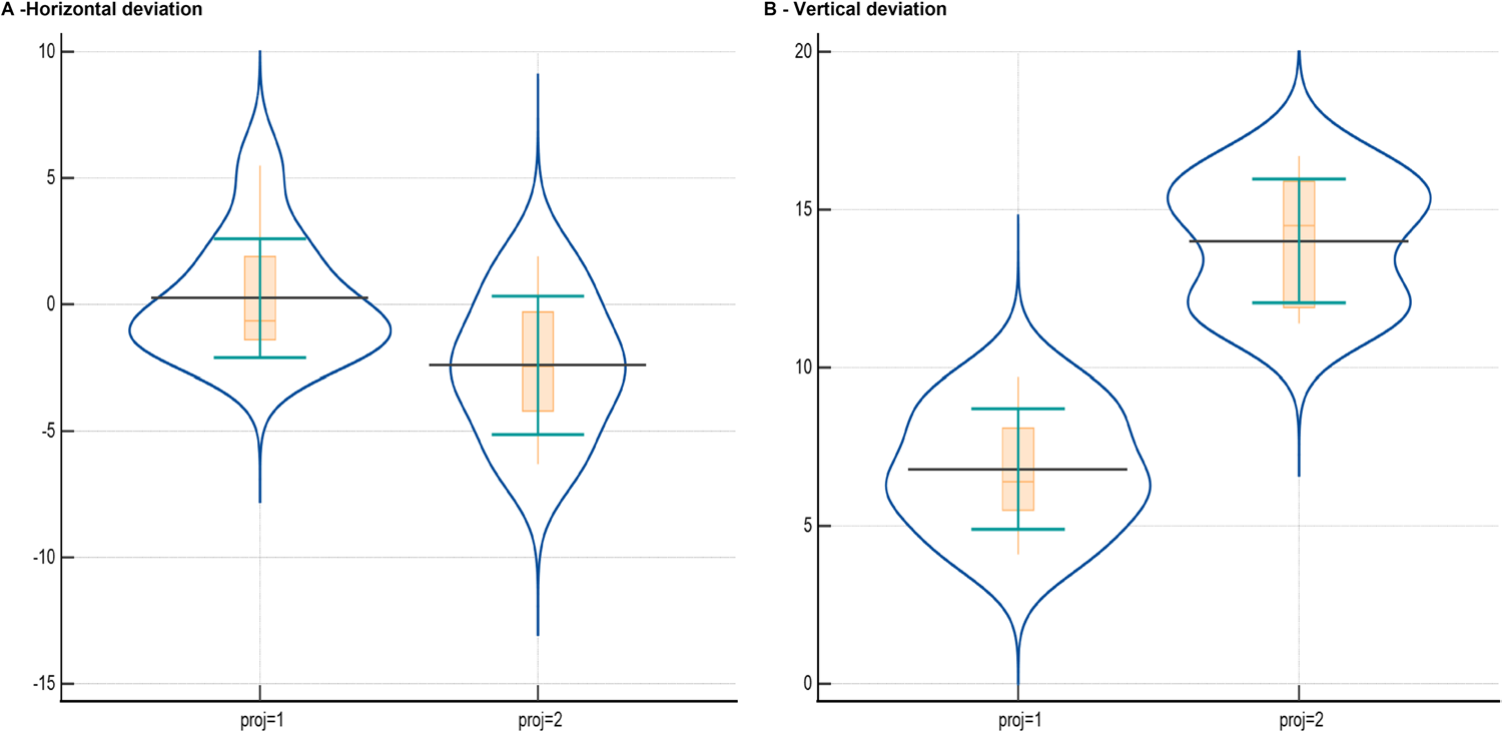
Horizontal and vertical deviation of the compounded round glass fragments. Violin plots displaying the horizontal (**A**) and vertical (**B**) deviation of the expelled compounded glass fragments for PEP (proj = 1) and FMJ (proj = 2) measured perpendicular to the windshield surface with black horizontal lines showing the mean values, orange lines within the boxplot resembling the median and green horizontal bars displaying the standard deviation (± 1 SD)

Comparison of the deviations of the expelled compounded glass fragments to the windshield is displayed in Fig. 6 and Table 5.

**Table 5:** Comparing deviation angles of the compounded glass fragments for FMJ and PEP. Statistically significant results are highlighted with a star. The comparison yielded statistically significant differences between PEP and FMJ regarding the vertical deviation of the expelled compounded glass fragments to the perpendicular of the windshield surface. Although the *p* value in the comparison regarding the horizontal deviation relating to the windshield is below 0.05, the difference is not of statistical significance due the small sample size being not sufficient for reliable test results in this comparison. The low significance of this difference can be explained by the approximation of the bullet flight path to the perpendicular of the windshield surface at the impact site. The difference in the comparison regarding the vertical deviation (with a difference of about 15° between the perpendicular of the windshield surface and bullet flight path) is statistically significant regardless of small sample size. The calculation of the required number of experiments is based on the assumption of alpha = 0.05 and beta = 0.1. Adjustment of *p* values was done using the Bonferroni method

Deviation to		*p*	*p* adjusted	Group 1 = PEP		Group 2 = FMJ		Sample size overall	
Parameter of comparison	Plane			*n*	Required *n*	*n*	Required *n*	*n*	Required *n*
Windshield	Vertical	.0002^*^	.0004^*^	10	4	10	4	20	8
Windshield	Horizontal	.0037	.0074	10	20	10	20	20	40

*FMJ* full metal jacket, *PEP* “Polizei Einsatz Patrone”—manufacturer’s designation of the deformation projectile

## Discussion

In the present study, shots in multiple experimental shooting series at laminated glass windshields showed reproducibly the formation of expelled round-shaped compounded glass fragments during projectile transition, which consisted of fine glass fragments connected by a punched sheet of the PVB interlayer. Hereby, fracture morphology allowed for unequivocal attribution of these round-shaped compounded glass fragments to two different types of 9 × 19 mm projectiles. Thus, it is possible to differentiate whether a bullet hole in the glass windshield was evoked by a perforating deformation projectile loaded in cartridges regularly used by German police or a commercially available round-nosed FMJ projectile.

In an experimental scenario, resembling a windshield angle of 15° being hit by a horizontally shot bullet round-shaped compounded glass fragment trajectory angles showed significant deviation from the expected right angle to the plane of the glass (Table 5; Fig. 6) [3–6, 8]. Expelled glass fragment trajectories had a tendency towards the bore axis of the gun barrel or rather the trajectory of the projectile. Based on the findings of this study, the extent of this reproducible deviation appears to be strongly dependent on the cartridge used. Whilst round-shaped compounded glass fragments originating from shots fired with the PEP 2.0 cartridge regularly showed trajectory angles between the right angle of the plane of the glass and the flight path of the projectile, trajectory angles of round-shaped compounded glass fragments originating from shots fired with the FMJ cartridge tended to approximate the flight path of the projectile.

For both combinations of cartridges and weapons, resulting expelled round-shaped compounded glass fragments were able to perforate cotton textile and penetrate up to 2.4 cm into ballistic gelatin. Textile defects were round-shaped and differed considerably from typical textile defects caused by smaller glass fragments expelled within the conical pattern at right angles of the plane of glass. The textile defects caused by expelled round-shaped compounded glass fragments were larger in diameter and may therefore be mistaken as defects resulting from deformed bullet penetration or a textile wrinkle formation in the bullet trajectory. Thus, findings have to be interpreted carefully, especially in the context of a reconstruction of the position of a victim at the moment a shot was fired. As pertains to the wounding potential, the observed penetration through skin surrogate and into ballistic gelatin indicates that expelled round-shaped compounded glass fragments may either cause potentially life-threatening injuries, e.g. affecting superficial blood vessels of the neck region, or that resulting injuries of tangential contacts may be mistaken as an additional grazing shot. Thorough cooperative multidisciplinary investigation and assessment of findings using the expertise of medicolegal, forensic ballistics and physiochemical experts should be sought to ensure an optimal result.

Despite the potential relevance of the injuries caused by expelled round-shaped compounded glass fragments for correct assessment of a given injury pattern in a specific case scenario, it has to be stressed that in the present study cartridges were fired exclusively with a 9 × 19 mm HK P30. Thus, distinct physical variables leading to projectile-dependent fragment morphology patterns and different trajectory angles will have to be identified in detail for alternative scenarios. Here, the distinct bullet design of both projectile types resulting in different contact surfaces interacting with the formation of fracture lines in glass appears to be an obviously decisive variable. Furthermore, parameters like yaw, the angle of attack, impact angle, bullet velocity and the properties of the laminated glass such as thickness and the characteristics of the plastic interlayer are likely to significantly influence the properties and behaviour of compounded glass fragments. For a better understanding of future case scenarios and to prevent misinterpretations of findings, trajectory angles and morphology of compounded glass fragments may ideally be assessed based on the reconstruction and physical testing of the respective constellations of weapons, cartridges and impact angles of laminated glass as an intermediate target.

Future research has to focus on the interdependence of the abovementioned parameters and resulting compounded glass fragments in different types of scenarios as pertains to weapons, cartridges and targets.

### Limitations

To address limited statistical power due to small sample size, the testing was complemented by estimation of type-I- and type-II error. These complementary analyses (“table statistics”) showed that the differences between FMJ and PEP regarding the vertical deviation measure were sufficient to reach statistical significance regardless of a rather small case number. Additionally, to minimise type-I-error inflation, the total number of tests was strictly restricted to assess the primary objective of this study.

## Conclusions

From this experimental series of shots at laminated glass windshield, the following conclusions can be derived:

1. After bullet penetration through laminated glass, in addition to a conically expelled spray of fine glass fragments, a round-shaped compounded glass fragment is formed and expelled at the exit site, consisting of fine glass fragments held together by a punched sheet of the PVB interlayer.
2. The fracture morphology of round-shaped compounded glass fragments may allow for the distinction of different bullet types perforating laminated glass.
3. The trajectory angles of round-shaped compounded glass fragments are reproducibly affected by the type of bullet penetrating the windshield and differ from the usually observed fine spray of glass fragments after bullet perforation through unlaminated glass.
4. The impact of round-shaped compounded glass fragments bears a higher wounding potential than the conically expelled spray of fine glass fragments.
5. Defects in textile layers caused by round-shaped compounded glass fragments can resemble those occurring after bullet penetration.
